# Receptor clustering and pathogenic complement activation in myasthenia gravis depend on synergy between antibodies with multiple subunit specificities

**DOI:** 10.1007/s00401-022-02493-6

**Published:** 2022-09-08

**Authors:** Natalie Rose, Sebastian Holdermann, Ilaria Callegari, Hyein Kim, Isabelle Fruh, Ludwig Kappos, Jens Kuhle, Matthias Müller, Nicholas S. R. Sanderson, Tobias Derfuss

**Affiliations:** 1 Department of Biomedicine, University Hospital Basel and University of Basel, Basel, Switzerland; 2 Neurologic Clinic and Policlinic and MS Center, University Hospital Basel, University of Basel, Basel, Switzerland; 3 Research Center for Clinical Neuroimmunology and Neuroscience (RC2NB), University Hospital and University of Basel, Basel, Switzerland; 4 Chemical Biology and Therapeutics, Novartis Institutes for BioMedical Research, 4002 Basel, Switzerland

**Keywords:** Myasthenia gravis, IgG_4_, Complement, Clustering, Human induced pluripotent stem cells, Live cell imaging

## Abstract

Myasthenia gravis is an autoimmune disorder defined by muscle weakness and fatigability associated with antibodies against proteins of the neuromuscular junction (NMJ). The most common autoantibody target is the acetylcholine receptor (AChR). Three mechanisms have been postulated by which autoantibodies might interfere with neurotransmission: direct antagonism of the receptor, complement-mediated destruction of the postsynaptic membrane, and enhanced internalization of the receptor. It is very likely that more than one of these mechanisms act in parallel. Dissecting the mechanisms of autoantibody-mediated pathology requires patient-derived, monoclonal antibodies. Using membrane antigen capture activated cell sorting (MACACS), we isolated AChR-specific B cells from patients with myasthenia gravis, and produced six recombinant antibodies. All AChR-specific antibodies were hypermutated, including isotypes IgG_1_, IgG_3_, and IgG_4_, and recognized different subunits of the AChR. Despite clear binding, none of the individual antibodies showed significant antagonism of the AChR measured in an in vitro neuromuscular synapse model, or AChR-dependent complement activation, and they did not induce myasthenic signs in vivo. However, combinations of antibodies induced strong complement activation in vitro, and severe weakness in a passive transfer myasthenia gravis rat model, associated with NMJ destruction and complement activation in muscle. The strongest complement activation was mediated by combinations of antibodies targeting disparate subunits of the AChR, and such combinations also induced the formation of large clusters of AChR on the surface of live cells in vitro. We propose that synergy between antibodies of different epitope specificities is a fundamental feature of this disease, and possibly a general feature of complement-mediated autoimmune diseases. The importance of synergistic interaction between antibodies targeting different subunits of the receptor can explain the well-known discrepancy between serum anti-AChR titers and clinical severity, and has implications for therapeutic strategies currently under investigation.

**Supplementary Information:**

The online version contains supplementary material available at 10.1007/s00401-022-02493-6.

## Introduction

Myasthenia gravis (MG) is a debilitating autoimmune disease associated with autoantibodies against components of the synapses between motor neurons and muscles (neuromuscular junctions, NMJ), making it one of the few autoimmune diseases in which the nature of the autoantigen provides an explanation for the symptoms. Various proteins can be involved, but four out of five patients [9, 44] have antibodies against subunits of the acetylcholine receptor (AChR). The receptor is a ligand-gated ion channel of four closely related subunits, alpha (α), beta (β), delta (δ) and epsilon (ε), each a four-pass transmembrane protein. Each receptor is a pentamer formed by two α, and one each of the other subunits [42]. There is inter-individual variation in the proportions of autoantibodies targeting the four subunits of the receptor [21, 43]. Action potentials arriving along the motor nerve result in the release of acetylcholine, which diffuses across the synaptic cleft of the neuromuscular junction, binds to the AChR and induces the opening of the channel, leading to depolarization and contraction of the muscle. Neural control of skeletal muscle is therefore completely dependent on the AChR, but how autoantibodies disrupt this process is not clear. Three mechanisms have been postulated, namely: direct blockade of the receptor, destruction of the receptor-bearing membrane by antibody-driven complement activation, and depletion of the receptors by antibody-mediated cross-linking and internalization [6, 7, 23, 40]. Sera from patients with anti-AChR-associated myasthenia gravis show evidence of all three of these mechanisms, in varying proportions [5, 28, 41].

Important advances have been made by studying whole sera or crude antibody preparations extracted from sera [17, 43], but understanding the relationship between antibodies and pathomechanisms requires examining the properties of individual patient-derived antibodies. For example, the isolation of antibodies against the muscle-specific kinase (MuSK), which are found in a small subset of myasthenic patients, has enabled the elucidation of their epitope specificity, and their effects on AChR clustering and MuSK phosphorylation [15, 40].

The isolation of autoantibodies against AChRs can be achieved by similar methods [25], but this approach requires that the antigen be prepared in a soluble form. In the case of AChR, this is complicated by the multi-membrane-pass, heteropentameric nature of the antigen. We therefore developed methods for isolating B cells specific for AChR from MG patients, using intact, membrane-expressed AChR as bait antigen, and examined the pathogenic potential of their anti-AChR antibodies in molecular mechanistic detail.

## Materials and methods

### Patients and healthy donors

Peripheral blood samples were collected from 12 healthy controls, six female and six male participants with an average age of 42, and 17 patients with clinically confirmed myasthenia gravis showing AChR-autoantibody RIA measurements above 0.5 nmol/l, with 6 female and 12 male participants and an average age of 62 (Supplementary Table 1).

Peripheral blood was drawn into S-Monovette tubes containing 1.6 mg EDTA per ml blood (01.1605.100) for isolation of PBMC, and into S-Monovette tubes with clot activator (01.1601.100, both from Sarstedt) for serum preparation. PBMC isolation and serum preparation were performed as previously described [1, 47]. PBMC were stored in liquid nitrogen until use, serum was stored at − 20 °C. The project was reviewed and authorized by the Ethikkommission Nordwest und Zentralschweiz.

### Plasmids and cell lines

TE671 rhabdomyosarcoma cells were obtained from ATCC (LGC, Wesel, Germany) and cultured in complete RPMI medium (10% heat-inactivated fetal calf serum (FCS), 100 units/ml of penicillin and 100 µg/ml of streptomycin; all from Gibco), at 37 °C in 5% carbon dioxide. β, δ, and ε subunits of AChR cloned into pcDNA3.1-hygro, and α subunit cloned into pEGFP-N1 for an intracellular eGFP tag were a gift from David Beeson [20] and used to transfect TE671 cells to produce TE-AChR-GFP. pCMV3 containing the open reading frame of human CD40 ligand was purchased from Sino Biological. TE cells were stably transfected, sorted for CD40L expression, and irradiated with 72 Gy for mitotic inactivation and kept frozen in liquid nitrogen until use. pUltra was purchased from Addgene (24,129).

### Identification of antigen-specific B cells using MACACS

AChR-specific B cells were isolated by the method described by Zimmermann et al. (2019). B cells were enriched from thawed PBMC by negative selection (Pan B cell isolation kit, human, Miltenyi). Five donors were selected from among anti-AChR-seropositive patients, on the basis of no recent immunosuppressive therapy (azathioprine within 6 months, glucocorticoids within 2 months, anti-CD20 ever). TE-AChR-GFP were labeled with cell trace blue (Thermo Fisher cat# C34568), and the extracellular AChR labeled with A647-conjugated α-bungarotoxin (α-BTX, B35450, ThermoFisher), then washed with complete RPMI medium. B cells were added to the adherent monolayer of labeled TE-AChR-GFP and incubated for 3 h. B cells were then retrieved and incubated for 20 min on ice with PE-conjugated anti-human CD69 diluted 1:100 in cold separation buffer. B cells were sorted on a FACSAria III Cell Sorter (BD Biosciences), gating on scatter to select live, single cells and on cell trace blue negative to exclude TE-AChR-GFP cells. Cells that were double positive for GFP and A647 were sorted into 1.5 ml Eppendorf tubes containing 700 µl RPMI-40 (RPMI with 40% FCS, 100 units/ml of penicillin, 100 µg/ml of streptomycin, and 50 ng/ml recombinant human IL-21 all from Gibco). The monoclonal antibody 8B4, an IgG1 specific for the hemagglutinin of Influenza A/California/07/2009 [47], was isolated by exactly the same process, from a healthy donor shortly after seasonal influenza immunization.

### Ex vivo B cell activation and high-throughput screening of single B cell supernatants

B cells were plated in flat-bottomed 384-well plates at a density of 1 B cell per well in the presence of 5000 irradiated TE-CD40L and 50 ng/ml recombinant human IL-21 in 75 µl RPMI-40. The outer wells of each plate were filled with 120 µl sterile H_2_O, plates were wrapped in aluminum foil to limit evaporation, and placed in a humidified incubator with 5% CO_2_ and 8% O_2_ at 37 °C for 12 days.

15 µl of B cell culture supernatant from each well was incubated with 10 µl separation buffer containing 5000 TE-AChR-GFP for 30 min at room temperature in the dark. Cells were washed and incubated with goat PE anti-human IgG at 1:200 (109-116-098), A647 anti-human IgA at 1:400 (109-605-011), and A594 anti-human IgM at 1:200 (109-585-129; all from Jackson ImmunoResearch), washed again and fixed in 4% PFA in PBS. Cells were acquired on a Cytoflex flow cytometer (Beckman Coulter).

### cDNA generation, Illumina sequencing and antibody preparation

After the screening, all but 10 µl of cell culture supernatant was removed and transferred to storage plates using a ViaFlo 384 pipetting apparatus (Integra). B cells were lysed in 20 µl of 15 mM Tris–HCl, pH 8.0 containing 0.5 U/µl of recombinant murine RNAse inhibitor (NEB, M0314L). Plates containing lysed B cells were stored at − 80 °C, plates containing supernatant at − 20 °C. RNA was isolated from lysed samples using the Quick-RNA MicroPrep Kit (Zymo). In brief, samples were thawed at room temperature, then mixed with 100 µl lysis buffer. 130 µl 100% ethanol was added and the mixture transferred to the column. After centrifugation, the column was treated with DNAse for 15 min at room temperature. The column was first washed with RNA prep buffer, then twice with RNA wash buffer before the RNA was eluted in 15 µl H_2_O. 4.5 µl RNA was used to generate cDNA using the SMART-Seq^®^ v4 Ultra^®^ Low Input RNA Kit for Sequencing by Takara following the manufacturer’s instructions at half the volume per reaction. Illumina library preparation (Nextera XT DNA Library Preparation Kit) and sequencing on a NexSeq500 (Illumina) were exactly as described by Callegari et al. [1].

Immunoglobulin gene sequences were extracted from the raw sequencing reads using custom scripts in R. Fastq files from the sequencer were aligned using the QasR and Rsamtools packages to an artificial mini genome containing the genomic sequences of constant regions from δ, µ, γ, α, and ε heavy chain genes, and κ and λ light chain genes, and the number of reads aligning to each were used to infer the classes and subclasses. Variable regions were reassembled using tools from the ShortRead and BioStrings Packages. Assembled variable regions were checked for V(D)J open reading frames using IgBlast (NCBI). DNA constructs encoding the inferred amino acid sequence from leader to several bases into the constant region were synthesized by IDT with restriction sites at the termini to enable in-frame cloning into pUltra plasmids already containing the appropriate constant regions. Plasmids were transfected into HEK cells to produce the encoded antibodies, and when more than one light chain and one heavy chain was found in the same well, all possible combinations were examined for AChR binding. Adherent HEK293T/17 (ATCC) were cultured in medium supplemented with 10% heat inactivated FCS and 100 units/ml penicillin and 100 µg/ml streptomycin (DMEM-10) in 12 or 6 well plates. Cells were transiently transfected with 1.5 µg plasmids encoding the heavy and light chain of a recombinant antibody in 1.5 ml DMEM-10 using 150 µl jetprime buffer and 3 µl jetprime reagent (PPLU114-07, Polyplus transfection). After 48–72 h, the supernatants were harvested and binding of antibodies to AChR was verified. Antibodies with the expected binding properties were then custom-manufactured according to our sequences by SinoBiological (Eschborn, Germany), purified by protein-A affinity chromatography, and their purity assessed by reducing and non-reducing SDS-PAGE gel electrophoresis. The antibody B12L described by Makino et al. [25], referred to in the nomenclature of the current work as aG_1_12, and the anti-influenza hemagglutinin antibody 8B4, were made by Sino Biological in IgG_1_ subclass. Sequences of variable regions of heavy and light chains for all antibodies whose cloning is described are shown in Supplementary Table 2. To produce the naturally occurring IgG_4_ bG_4_02 as an IgG_1_, the heavy constant gamma 4 region was replaced with a construct encoding the heavy constant gamma 1 (Uniprot accession P01857.1).

### Flow cytometry of antibody binding, complement activation, and epitope determination

HEK cells transfected with the human adult AChR, incorporating an α-subunit fused with GFP (AChR-GFP), were used for flow cytometric assays. 80,000 cells diluted in 40 µl of DMEM-10 were seeded in a U-bottomed 96-well plate. For the antibody binding assay, 10 µl of serum or antibody was added and incubated for 30 min on ice. After three washing steps with cold PBS, bound antibody was labeled with DyLight™ 405 donkey anti-human IgG (JIR: 115-295-166) in a 1:200 dilution and finally analyzed via flow cytometry. In the case of the in-vitro complement activation assay, the mix of HEK cells with antibodies or serum was supplemented with 10 µl of human serum as source of complement components. Cells were incubated for 2 h at 37 °C and 5% carbon dioxide. After three repeated washing steps with PBS, complement activation were tested by labeling of C3 complement component deposition with a mouse monoclonal anti-complement C3/C3b/iC3b antibody (BioLegend Cat. No. 846402) for 30 min on ice. Following another three washing steps, secondary antibodies Rhodamine Red™-X goat anti-mouse IgG (JIR: 115-295-166) and DyLight™ 405 donkey anti-human IgG (JIR: 115-295-166) were used for labeling. Finally, after 20 min of incubation on ice and two rounds of washing with PBS, data for a target of 10,000 cells were acquired on a Cytoflex cytometer (Beckman Coulter), stored as.fcs files and analyzed using FlowJo (Becton Dickinson). To determine epitope specificity of the human-only anti-AChR antibodies, HEK cells were transfected with four plasmids, one encoding a human subunit, and three encoding rat orthologs of the other three subunits. Labeling and flow cytometry were otherwise the same as above. To determine epitope specificity by competitive blocking, HEK cells transfected with AChR-GFP were first incubated for 30 min with 2 µg/ml of a commercial, monoclonal, subunit-specific antibody (α: mAb35, BioCell; β, BioRad, cat MCA1329GA), washed once, and then labeled and analyzed as above.

### Human induced pluripotent stem cell (iPSC) generation and maintenance

The iPS line 90/1.2 (Invitrogen #C-013-5C) was derived from adult human dermal fibroblast lines from Invitrogen. Fibroblasts were reprogrammed using the CytoTune-iPS Reprogramming Kit (Invitrogen, cat-#A1378001). All cell lines were checked for normal karyotype and pluripotency. iPS cells were cultured on laminin 521 (Biolamina, cat-# LN521) coated dishes in mTeSR plus (Stem Cell Technologies, cat-# 05825) supplemented with 1% Pen/Strep (P/S) (ThermoFisher, cat-# 15070-063). Cells were dissociated with TrypLE (Gibco, cat-# 12604-013) every 3–4 days and plated at a density of 20,000/cm^2^ in the presence of 10 μM ROCK inhibitor (Calbiochem, cat-# Y-27632). The medium was replaced every other day.

The fast green fluorescent calcium indicator GCaMP6f open reading frame [2] was placed under the control of the CAG promoter, with a puromycin resistance gene and cloned into an AAVS1-targeting vector [12]. After co-transfection of the targeting vector with a Cas9 construct [34] and a guide (gtc acc aat cct gtc cct ag) against AAVS1 into a doxycycline-inducible MyoD iPS cell line, colonies were picked after puromycin (1 µg/ml) (Gibco, cat-# A11138-03) selection. Correct integration to the AAVS1 locus was validated via PCR analyses.

Human Ngn2 cDNA was synthesized according to sequences available from the Ensembl database (Ensembl Gene ID ENSG00000178403 or accession number NM_024019.3) and cloned under the control of TRE tight (Tetracycline Response Element) promoter in a PiggyBac/Tet-ON all–in-one vector. This vector contains a CAG rtTA16 cassette allowing constitutive expression of Tet-ON system and an Hsv-tkNeo cassette for generation of stable IPS clones. Differentiation to neurons was performed as reported previously [32]. Briefly, iPS cells were plated on Matrigel-coated cell culture plate in DMEM/F12 (ThermoFisher, cat-# 31331) supplemented with 2% B27 (ThermoFisher, cat-# 17504-044) and 1% N2 (ThermoFisher, cat-# 17502-048), 10 ng/ml of Human Epidermal Growth Factor (hEGF) (ThermoFisher, cat-# PHG0315), 10 ng/ml of basic human Fibroblast Growth Factor (hFGF) (ThermoFisher, cat-# CTP0263) and 1% penicillin/streptomycin (P/S) containing 10 µM ROCK inhibitor for 1 day and 1 µg/ml doxycycline (Sigma, cat-# D9891) for 3 days. Human Myoblast Determination Protein 1 (MYOD1) cDNA was synthesized according to Accession number NM_002478.5, cloned into the inducible vector and transfected into iPS cells as above. For differentiation, cells were cultivated in KSR Medium composed of Minimum Essential Medium α (ThermoFisher, cat-# 12571063), 5% Knockout™ serum replacement (KSR) (ThermoFisher, cat-# 10828028), 1% P/S, 55 µM β-mercaptoethanol (ThermoFisher, cat-# 21985023) and supplemented with 1 µg/ml doxycycline for 3 days to generate myoblasts. These myoblasts were further amplified in KSR Medium containing 20 ng/ml hFGF (ThermoFisher, cat-# CTP0263) for an additional 4 days in the same medium as described above.

40,000 myoblasts were seeded in a laminin-coated 384-well plate in KSR medium supplemented with ROCK inhibitor and doxycycline. One day later, medium was switched to myoblast differentiation medium: DMEM F12-Glutamax (Gibco, cat-# 31331028) with 5% FBS (HyClone, cat-# SH30070.02), 0.2% ITS (BD, cat-# 354351, 0.1% BSA (Sigma, cat-# A1595), 1% P/S, CHIR99021 (2uM) (Sigma, cat-# 1046), Dorsomorphin (1 uM), (Stemgent, cat-# 04_0024), Dibutyryl-cAMP (1 mM) (Sigma, cat-# D0627). Three days later, iNGN2 neurons, differentiated for 3 days in proliferation medium, were plated on top of myotubes in a 1:1 ratio and further differentiated in neuronal differentiation medium composed of NeurobasalTM Medium (ThermoFisher, cat-# 21103049), supplemented with 2% B27, 1% N2, 1% P/S, 10 ng/ml of human BDNF, GDNF and NT3 (R&D Systems, cat-# 248-BD, 212-GD and 267-N3, respectively) for approximately 3 weeks at 37 °C in 5% CO_2_. Medium was replaced every 2–3 days. MAP4K4 inhibitor (i.e. GNE-495) was added at 5 µM between day 5 and 14. 2 µM Cytosine β-d-arabinofuranoside (AraC) (Sigma, cat-# C1768) was added at day 5–12 to protect the culture from proliferating cells.

### Functional drug screening system read out from human iPSCs

Sera or antibody diluted in neuronal differentiation medium was added after about 3 weeks to the co-culture and was incubated for 3 h. For FDSS, medium was replaced by Ca^2+^ buffer (50 ml HBSS 10x (ThermoFisher, cat-# 14065049), 10 ml HEPES 1 M (ThermoFisher, cat-# 15630056), 440 ml dH_2_O and 375 µl CaCl_2_ (1 M). After 40 min, all plates were read by FDSS/µCELL Functional Drug Screening System (Hamamatsu, cat-# C13299) after injection of 0.5 µM AMPA (Sigma, cat-# A6816). Analysis was done with Hamamatsu software.

### Live cell imaging of cluster formation

HEK cells seeded in an 8-well chambered coverslip (Ibidi 80826) were transfected with the human AChR subunits α-GFP, β, δ, and ε, and allowed to express the receptor overnight at 37 °C in 5% carbon dioxide. On the following day the chambered coverslip was placed in a humidified chamber with controlled temperature and carbon dioxide concentration for imaging with a Nikon A1R confocal microscope. A 40 ×, 0.6 NA air objective was used and stacks of 8 planes spanning the focal plane of the cells were continuously acquired over the course of 10 min. Two cycles of such image acquisition were performed per well. The first cycle after a 10 min incubation with α-BTX, and the second after a change of medium and addition of single or combinations anti-AChR antibodies. Thereby two data sets of confocal images were generated, one showing the AChR distribution on the cell surface before and the other after antibody addition.

### Passive transfer myasthenia gravis in Lewis rats

Procedures involving live animals were reviewed and permitted by the cantonal animal research commission. Four-week-old female Lewis rats obtained from Janvier Labs received only one intraperitoneal injection of PBS, or a human monoclonal antibody dissolved in PBS under isoflurane anesthesia. Monoclonal antibodies included 8B4, bG_4_02, bG_4_02-G_1_, aG_1_01 or a combination of bG_4_02-G_1_ and aG_1_01. Each rat received 4 mg/kg of total antibody, i.e. 4 mg/kg of each single antibody, or 2 mg/kg of each antibody in the case of combinations. For the animal experiments with a behavioral endpoint the rotarod performance as well as weight loss and clinical score were assessed every 12 h. Each animal was scored based on guidelines for pre-clinical assessment of anti-AChR antibody induced myasthenia gravis as reported elsewhere [18, 25]. The clinical scale was defined as follows: 0, no weakness; 1, first signs of a weakened grasp and fatigable after several trials; 2, incomplete paralysis of hind limbs and clinical signs of weakness; 3, severe clinical signs of weakness with no ability to grip, hindlimb paralysis and moribund; 4, death. Animals were euthanized if they reached clinical score 3 with severe muscle weakness. One animal was found dead already at the 24 h testing time point. Rats were examined and trained on the rotarod machine to establish the baseline starting 72 h prior to injection, and 48 h after the injection all rats were sacrificed by transcardial perfusion fixation with 4% paraformaldehyde (PFA). Subsequently, the gastrocnemius muscle, the soleus muscle and the diaphragm were isolated and stored in 4% PFA until further analysis. In the second experiment for investigating complement activation, the same procedure was performed but the animals were sacrificed at 12 h.

### Immunofluorescent labeling of rat muscle sections and iPSC cocultures

The gastrocnemius muscle stored in 4% PFA was cryoprotected in an ascending concentration series of 10%, 20% to 30% sucrose-PBS solution. After snap-freezing, the rat muscles embedded in OCT (CellPath) on dry ice were cut into 30 µm thick cryosections with a Leica cryostat. Rat muscle sections stored at − 80 °C for immunofluorescent labeling were thawed for 10 min at room temperature, washed twice with PBS and incubated with 0.1 M glycine in PBS for another 10 min. Following a 10 min incubation with ice-cold 80% methanol and two PBS washing steps, the rat muscle sections were blocked with a solution containing 1% FCS, 3% BSA and 0.3% Triton X-100 in PBS for 1 h. Primary antibodies to label rat C3 complement deposition (mouse anti-rat IgG, clone 12E2, Novus Biologicals, NBP1-05140) and SV2A (rabbit anti-rat IgG, Novus Biologicals, NBP1-82964) were diluted in blocking solution for a final concentration of 1 µg/ml and 0.3 µg/ml, respectively and incubated for 16 h at 4 °C in a humid chamber at 4 °C. After washing with PBS, the sections were soaked with goat anti-mouse IgG (H + L) Rhodamine Red™-X (Jackson ImmunoResearch 115–295-166, final concentration 4 µg/ml), goat anti-rabbit IgG Alexa Fluor 488 (Jackson ImmunoResearch 111-545-144, final concentration 4 µg/ml) and α-BTX (ThermoFisher B35450, final concentration 1 µg/ml) diluted in blocking solution for 16 h at 4 °C. Finally, the muscle sections were washed with PBS, labeled in PBS-containing 1 µg/ml DAPI, mounted with Fluoromount-G and sealed with a coverslip. Depending on the analysis, images were acquired with a Nikon A1R scanning microscope with a 60 ×, 1.4 NA OI objective using voxel dimensions set to Nyquist sampling, a Nikon Crest V3 spinning-disc confocal microscope with a 40 ×, 0.95 NA air objective or a Nikon Eclipse TI2 equipped with a 20 ×, 0.75 NA air objective. For investigating human antibody binding at the neuromuscular junction, the sections were not treated with primary antibodies, but directly incubated with α-BTX and goat anti-human IgG FITC (Jackson ImmunoResearch 109-096-098, final concentration 4 µg/ml). Further processing and image acquisition were performed as described for analyzing complement deposition.

Cocultures of hiPSC-derived cells were fixed in 4% paraformaldehyde at room temperature for 10 min, followed 3 × DPBS (Sigma, cat-# 14190) washings, 5 min each. DPBS supplemented with 0.1% Tween and 2.5% BSA or for permeabilization 0.1% Triton X-100 and 1% BSA (blocking) was used for primary antibody labeling, overnight at 4 °C. The following antibodies were used: NF-200 (Abcam, cat-# Ab72996); α-Actinin (Abcam, cat-# Ab9465), Tuj1 (cat-# Sigma T8660); MAP2 (cat-# Abcam Ab5392). After three washing steps with DPBS, cells were incubated for 1 h with secondary antibodies (Invitrogen). Afterwards, cells were washed in DPBS and incubated with DAPI for 10 min. Ibidi mounting medium (IBIDI, cat-# 50001) was added to the wells and stored at 4 °C. Images were acquired with LSM900 microscope using Zen 3.2 (Blue edition) software.

### Image analysis

Nikon NIS-Elements software and Fiji based on ImageJ2 were used for image analysis. Based on the SV2A signal a mask was designed to define the area of the NMJ. The α-BTX fluorescence signal intensity inside this mask was measured and the ratio to the colocalizing SV2A calculated. In total, eight images from three different gastrocnemius cryosections per animal were analyzed amounting to more than 600 NMJs tested for each injected group. For complement activation, the fluorescence signal intensity of C3 deposition was measured for a minimum of 100 NMJs per rat and four rats per condition. The structural analysis was performed on high magnification image stacks of 15 individual NMJs per animal. Initial processing included the generation of maximum intensity projections, the separation of the α-BTX channel, the reduction of background and selection of a threshold based on the signal intensity. From the resulting image, the number of individual objects, object area, and mean fluorescence intensity were measured. The analysis of the live cell imaging is based on the same approach. For each image acquired over the course of the 20 min of imaging, a threshold level of α-BTX signal was calculated with the RenyiEntropy function of ImageJ, and areas of signal exceeding this threshold, of an area between 0.8 and 10.0 square microns, were defined as “clusters”, whose area and number were extracted using an automated macro in Fiji. R scripts were applied to calculate the total area, mean area and total number of clusters in each image, and plot the data as bubble charts.

## Statistics

GraphPad Prism was used for statistical analysis. Prior to performing statistical tests, the data were tested for normal distribution with the Normality and Lognormality test. Quantification of the AChR content of NMJs was compared using a One-Way ANOVA with a multiple comparison corrected according to Tukey. For C3 deposition and structural parameters either a Two-Way ANOVA or a Kruskal–Wallis test with a Dunn’s multiple comparison was applied depending on the normal distribution of the dataset. Differences between MG patients and healthy volunteer serum in signal transmission in the iPSC-derived neuromuscular model were tested using a two-way ANOVA with a multiple comparison corrected according to Tukey.

## Results

### Serum from patients with MG inhibits neuromuscular signal transduction and induces complement activation

**Fig. 1:**
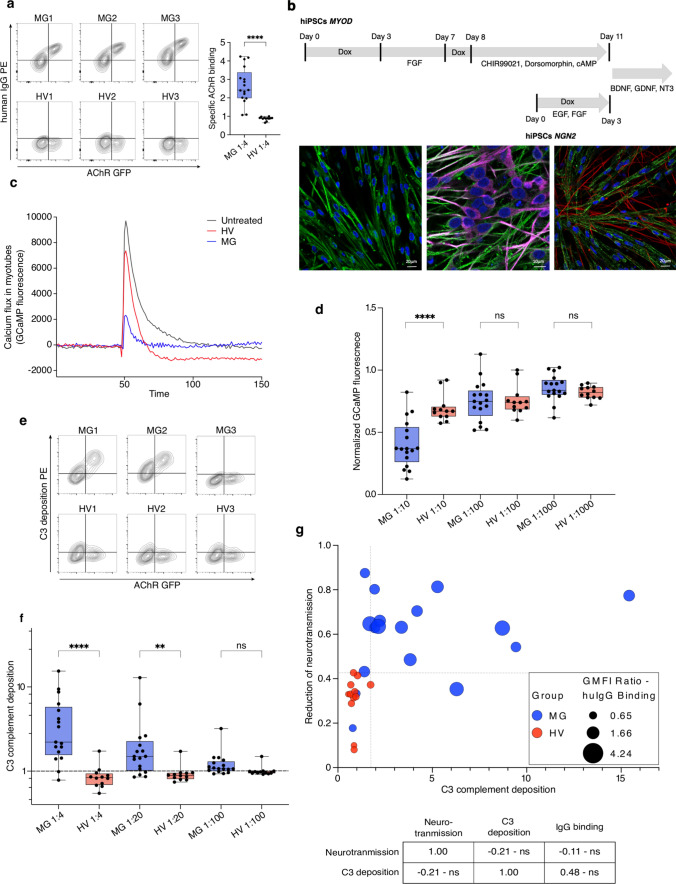
Inhibition of neuromuscular signal transmission and induction of complement activation by serum from patients with MG. **a** Live cell flow cytometry of specific antibody binding to AChR. Sera from 3 MG patients and 3 healthy controls were incubated with TE671 cells expressing human AChR-GFP. The horizontal axis depicts the fluorescence intensity of GFP and the vertical axis the fluorescence intensity of the anti-human IgG secondary antibody. The box and whisker plot on the right shows the specific AChR binding (the geometric mean fluorescence intensity (GMFI) ratio, i.e., the fluorescence signal for anti-IgG immunofluorescence on AChR-transfected cells divided by the signal on non-transfected cells) in serum from 17 MG patients and 12 healthy control sera. Demographic information about these donors is shown in Supplementary Table 1. Results shown in d and f come from the same donors. The difference between the groups was significant (****p < 0.0001) by two-tailed Mann–Whitney *U* test. **b** The hiPSC-derived neuromuscular system. Schematic figure shows timeline of co-culture generation. hiPSC MYOD and hiPSC NGN2 cells were treated with doxycycline and growth factors to direct their differentiation to muscular and neuronal phenotypes, respectively. Co-culture started on day 13 with iND3 plating on iMD13. Fluorescence photomicrographs below the schematic timeline show cocultures labeled for neuronal or muscular markers. Shown in the left panel is a myotube culture before iND3 neurons were added. The typical muscular striations are visible after labeling the myogenic marker α-actinin (green). The middle panel shows a pure neuronal culture at 10 days labeled with markers for mature neurons, MAP2 (green) and Tuj-1 (pink). The right panel shows an image of the co-culture after 3 weeks. Myotubes are labeled for α-actinin (green) and neurons for NF200 (red), another marker for neurons. DAPI (blue) labels nuclei. See also live cell imaging of co-culture (Supplementary Video 1). **c** Effect AChR blockade on signal transduction in the iPSC-derived neuromuscular model. Myotubes express GCaMP, allowing intracellular calcium to be monitored by fluorescence quantification. The horizontal axis displays the time, with the AMPA stimulus added at time 50. The vertical axis shows the fluorescent signal corresponding to intracellular calcium in myotubes. The black trace is the signal in cocultures without serum added. The red trace shows the signal in cultures to which serum from a healthy donor was added. The blue trace shows the signal after addition of serum from a patient with MG. **d** The effect of MG serum on acetylcholine-dependent calcium flux in myotubes of the hiPSC-derived neuromuscular system. The vertical axis shows the normalized cholinergic transmission (peak height of the GCaMP calcium signal measured as explained in c, divided by the mean value of untreated wells). Each point shows the mean of 3 repeat measurements with one serum. The assay was performed at 3 different dilutions of serum from patients with myasthenia gravis (MG) or from healthy volunteers (HV) as shown below the horizontal axis. Differences between results using sera from the two sources were compared using a two-way ANOVA with multiple comparison correction according to Tukey (****p < 0.0001). **e–f** Antigen-dependent complement activation by MG sera. **e** Sera from 3 MG patients and 3 healthy controls were incubated with HEK cells expressing human AChR-GFP, and complement deposition was measured with an anti-C3 antibody by flow cytometry. The vertical axis of each contour plot shows the anti-C3 signal, and the horizontal axis the AChR-GFP fluorescence. The top row shows results for sera from MG patients, and the lower row results from HV controls. **f.** Sera from 17 donors with MG and 12 healthy donors were measured as shown in e. The vertical axis shows C3 deposition (GMFI ratio, i.e., the fluorescence signal for anti-C3 immunofluorescence on AChR-transfected cells (the right side of each plot in e), divided by the signal on non-transfected cells (the left side of each plot in e). Differences between conditions were compared by Kruskal–Wallis test, followed by Dunn’s multiple comparison (**p = 0.0063, ****p < 0.0001). **g** Relationships between anti-AChR IgG binding, complement activation, and signal transmission blockade. For each serum sample (healthy, “HV”, red symbols; or myasthenic, “MG”, blue symbols) complement activation measured by C3 deposition as shown in **e**–**f** is plotted against the reduction of cholinergic transmission as measured in **c**–**d**, with symbols whose size represents IgG binding to AChR, as shown in **a**. The table below the plot shows the Spearman’s rank correlation coefficients and the *p* values calculated by two-tailed permutation test. None of the correlations between the three pairs of variables was significant

To enable examination of antibodies interacting with antigen in its native conformation, we developed a suite of techniques based on expression of the native human AChR on live cells. Antibody binding was measured by flow cytometry on AChR-transfected cells. AChR-binding antibodies of the IgG class were detected in all patients who were AChR-seropositive by the clinical radioimmunoassay, and diagnosed with clinically definite MG (Fig. 1a, Table 1).

**Table 1:** Patient and donor information, of whom serum was tested individually to provide representative results shown in Fig. 1a, e

Participant	Sex	Age	Diagnosis	Thymus status	Disease onset	Besinger Score	Immuno-therapy	RIA AChR antibody titer (nmol/l)
MG1	M	63	Ocular AChR MG	No thymoma	02/2018	6	None	16.5
MG2	M	35	Generalized AChR MG	Thymoma	06/2018	8	None	27.4
MG3	F	39	Generalized AChR MG	Slight thymic hyperplasia	2014	6	None	366
HC1	F	38	Healthy control	–	–	–	–	–
HC2	F	36	Healthy control	–	–	–	–	–
HC3	M	44	Healthy control	–	–	–	–	–

Furthermore, anti-AChR specific B cells were isolated from these 3 MG patients and used for the production of recombinant monoclonal anti-AChR antibodies. Patients who provided serum and the B cells from which monoclonal antibodies were isolated, and healthy controls who provided serum. Comparisons of AChR binding and AChR-dependent complement activation between patients and controls are shown in Figs. 1a, e, respectively

AChR antagonism was assessed by measuring the reduction in acetylcholine-induced calcium flux in a human iPSC-derived neuromuscular system. This system consisted of neuronal cells and myotubes generated by overexpression of MYOD1 as the myogenic and NGN2 as neurogenic master regulators, enabling in vitro modeling of the neuromuscular junction (Fig. 1b). Cholinergic neurotransmission was monitored by calcium imaging in myotubes stably expressing GCaMP. Due to spontaneous electrical activity, individual myotubes occasionally depolarize, resulting in brief peaks of fluorescence (see Supplementary Video 1). Following stimulation of the presynaptic neurons with α-amino-3-hydroxy-5-methyl-4-isoxazolepropionic acid (AMPA), all myotubes in the culture depolarize and fluoresce together, yielding a strong signal that can be recorded and quantified. Any reduction in responsiveness of the myotubes to acetylcholine release from the stimulated neurons is observed as a reduction in the magnitude of this signal (Fig. 1c). Compared to sera from healthy donors, sera from patients with MG impeded neurotransmission from neurons to myotubes significantly (Fig. 1d). Direct AChR antagonism by serum constituents was independent of complement, because heat-inactivated sera showed the same results as fresh sera (Supplementary Fig. 1).

Antigen-dependent complement activation was measured by incubating cells expressing AChR, or untransfected control cells, with sera from patients and healthy controls, and measuring C3 deposition on the cell surface by flow cytometry (Fig. 1e). Sera from patients with myasthenia gravis induced complement activation specifically on AChR-transfected cells, while sera from healthy volunteers did not. At dilutions up to 1:20, this difference was significant (Fig. 1f).

Sera from the majority of patients (65%) evinced both complement activation and receptor antagonism, but neither parameter was significantly correlated with total specific antibody binding, or with each other (Fig. 1g). Receptor antagonism in 3/17 patients, (18%), and complement deposition in 5/17 (29%) were within the range of the healthy controls. In two patients (11%), both values were in the normal range. These patients also had minimal total AChR-specific antibody binding (the two lowest values in Fig. 1a).

### Isolation of anti-AChR specific B cells by MACACS

**Fig. 2:**
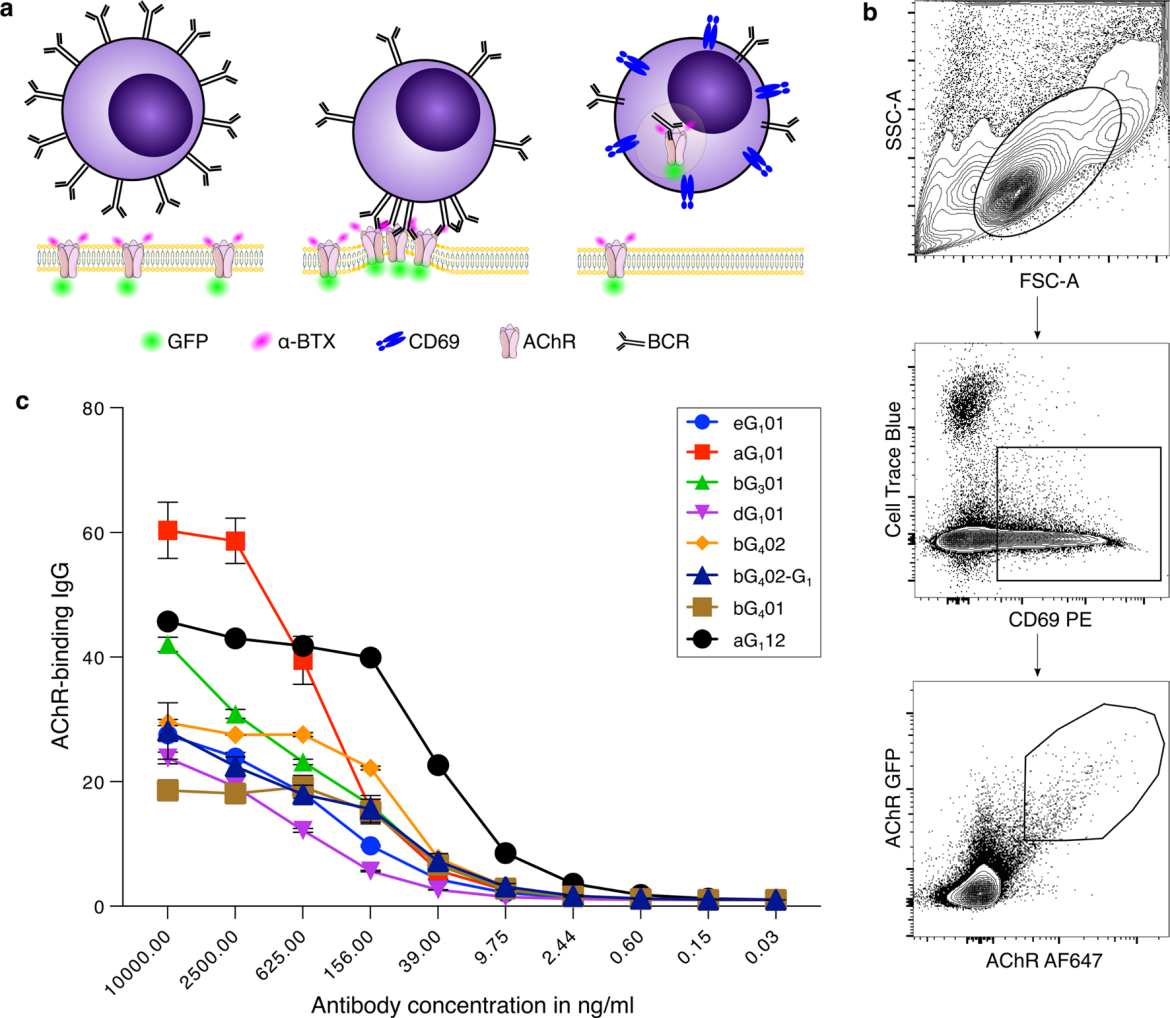
Isolation of AChR-specific B cells from MG patients by MACACS. **a** Schematic illustration of membrane antigen capture by a B cell. AChR-specific B cells recognize their cognate antigen in the membrane of a live cell, bind and extract it. This process leads to B cell activation, CD69 upregulation, and the acquisition of GFP signal. **b** Representative FACS plots showing the gating for B cells enriched by MACACS. The first plot shows forward and side scatter, the second plot shows the cell trace blue label used to exclude antigen donor cells, plotted against CD69. The third plot shows two markers used to detect antigen-specific cells, i.e., AChR-GFP, and α-BTX. Cells in the polygonal gate in the third plot were sorted and used to prepare monoclonal antibodies, as described in Materials and Methods. **c.** Binding of monoclonal antibodies to AChR. Serial dilutions (shown on the horizontal axis) of each of the six monoclonal antibodies prepared from B cells isolated as above were incubated with AChR-GFP expressing HEK cells and specific binding calculated as GMFI ratio of anti-human-IgG signal on AChR-expressing cells divided by signal on non-transfected cells. As a positive control aG_1_12 antibody described by Makino et al. [25] was used, and we also included the antibody bG_4_02 artificially switched from IgG_4_ to IgG_1_ (bG_4_02-G_1_)

Following the observation that patient sera mostly contain activities mediating both receptor antagonism and complement activation, it became apparent that investigation of either mechanism would require the isolation of monoclonal antibodies. To this end, we used the method of membrane antigen capture activated cell sorting (MACACS) described by Zimmermann et al. [47]. This method exploits the ability of live B cells to extract their cognate antigen from cell membranes, becoming highly activated in the process (Fig. 2a, b). By screening PBMC from 5 seropositive donors by this method, we isolated six AChR-specific antibodies from three donors with myasthenia gravis (Table 2). The antibodies, of classes IgG_1_, IgG_3_, and IgG_4_ are somatically hypermutated (7–19 amino acid replacements per heavy chain), and include two members of one expanded clone from one donor (Table 2). As an approximate indicator of affinity, we plotted AChR-specific binding at a range of concentrations and extracted the concentration required for half maximal specific binding (EC50), yielding values between 58 and 783 ng/ml (Fig. 2c).

**Table 2:** Antibody characteristics; list of anti-AChR specific antibodies isolated from the peripheral blood of MG patients

Antibody ID	Patient ID	Isotype	Aa replacements in V region	CDR3 aa length	α-subunit block (%)	β-subunit block (%)	Rat cross-reactivity	Subunit specificity
aG_1_01 1J7	MG3	IgG1	19	23	66	1	Yes	α
		κ	16	10				
bG_3_01 3I3	MG2	IgG3	11	25	3	68	No	β
		k	2	5				
bG_4_01 5D2^a^	MG1	IgG4	9	17	26	65	No	β
		λ3	3	9				
bG_4_02 6J2^a^	MG1	IgG4	12	17	16	66	Yes	β
		λ3	7	9				
bG_4_02-G_1_ 6J2 IgG1	MG1	IgG1	12	17	17	71	Yes	β
		λ3	7	9				
dG_1_01 5H10	MG1	IgG1	7	17	42	15	No	δ
		κ	7	9				
eG_1_01 2M18	MG1	IgG1	11	9	17	10	No	ε
		κ	1	9				

^a^Clonally related with shared use of H and L chain V(D)J combinations

Antibody binding experiments with HEK cells expressing the rat AChR showed that two patient-derived antibodies recognize receptors from both species, and four recognized only the human receptor. This enabled us to elucidate the subunit specificity of the human-only antibodies using chimeric receptors of mixed human and rat subunits (Supplementary Fig. 2) assuming that replacement of the target subunit with the human ortholog would confer binding on the otherwise non-binding rat receptor. To confirm these results, and to identify the targets of the rat- and human-reactive antibodies, we examined competitive blockade of the patient-derived antibodies by commercial monoclonal antibodies of known subunit specificity. The combined results from the two approaches suggested that one patient-derived monoclonal antibody targeted the α subunit, three antibodies the β, and one each the δ, and ε (Table 2). Based on the subclasses and the deduced epitope specificity, antibodies were assigned names reflecting these properties, e.g., aG_1_01 is the first α-binding antibody of subclass IgG_1_ (Table 2).

### Single antibodies have limited effect on pathomechanisms

**Fig. 3:**
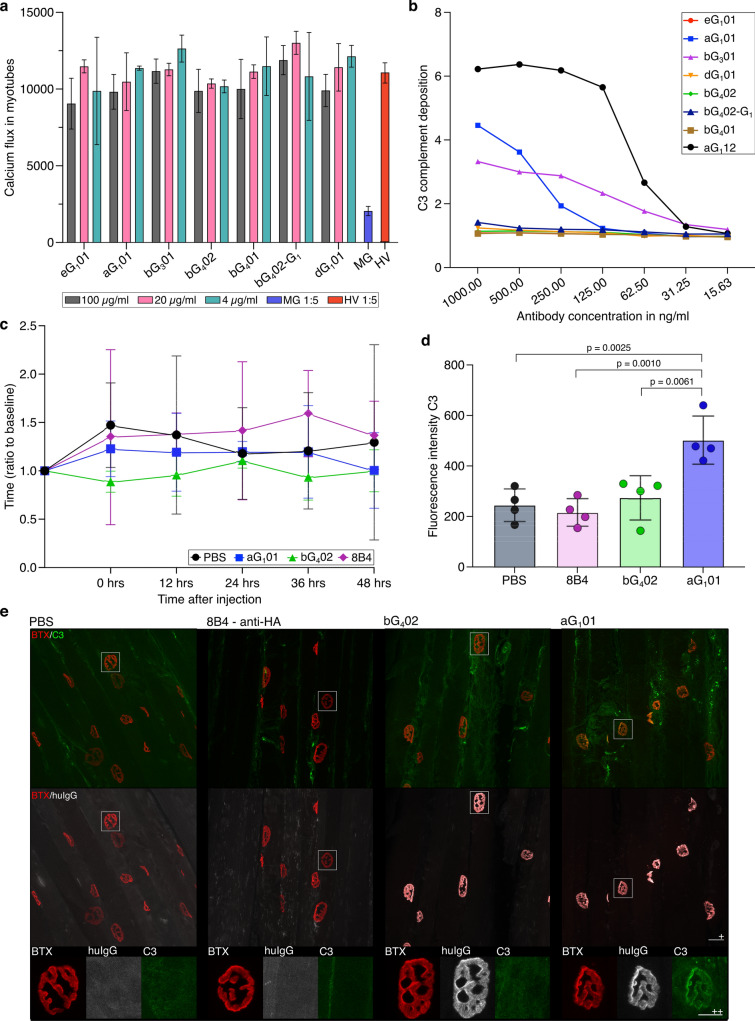
Pathogenic potential of single monoclonal AChR-specific antibodies derived from B cells from MG patients. **a** Effect of monoclonal AChR-specific antibodies on cholinergic transmission in the iPSC derived neuromuscular assay. Co-cultures of iPSC-derived neurons and myotubes were incubated for 3 h with the indicated concentrations of monoclonal antibodies, and the effect on AMPA-induced cholinergic signaling was assessed as in Fig. 1c, d. Vertical axis shows the acetylcholine-dependent calcium flux in myotubes as measured by GCaMP signal after AMPA stimulation. Bars show mean values from three replicates with each of the antibodies indicated below the horizontal axis, and the error bars show the SD. Sera from a patient (MG) or a healthy volunteer (HV) diluted 1:5 were included as positive and negative controls. **b** Antigen-dependent complement deposition on live cells measured by flow cytometry. Cells expressing AChR-GFP and untransfected control cells were incubated with serial dilutions of single AChR-specific monoclonal antibodies and supplemented with human serum as source of complement. Activation of the complement cascade was assessed by flow cytometry analysis of C3 deposition visualized with fluorescent secondary antibodies. The GMFI ratio was calculated as described in Fig. 1f. In vivo investigation of pathogenicity of single antibodies. **c** Passive transfer myasthenia gravis in rats. Graph shows rotarod performance of animals given PBS vehicle (black lines and circles), the anti-hemagglutinin IgG_1_ control antibody 8B4 (purple lines and diamonds), the anti-α IgG_1_ aG_1_01 (blue lines and squares) or the anti-β IgG_4_ bG_4_02 (green lines and triangles). The horizontal axis shows the time after antibody administration, and the vertical shows the relative time animals remained on the rod (ratio of time on rod at measurement, divided by average time for the three trials before antibody administration) at each of the five timepoints after injection. Each point is the mean of four animals in each condition. Error bars show the standard error of the mean. No significant difference was observed in mean times between groups at any time point (two-way ANOVA with repeated measures, *n* = 4 per group). **d** Complement deposition at neuromuscular junctions. Cryosections from gastrocnemius muscles from the animals whose behavior is shown in c were labeled with α-BTX to visualize the AChR, and immunolabeled with a mouse anti-rat C3 antibody to reveal complement deposition. C3 labeling was quantified at at least 100 NMJ per animal, and the vertical axis shows the mean value per animal across NMJs. *P* values were calculated by one-way ANOVA, followed by Tukey's test. Error bars show standard deviation. An analogous figure, with values for individual NMJ is shown in Supplementary Fig. 4. **e** Histopathological impact of monoclonal antibody administration. Cryosections as described in d were immunolabeled, in addition to C3(green), for human IgG (white), and α-BTX (red). From left to right, the first image comes from a rat injected with PBS, the second from a rat injected with 8B4, the third bG_4_02 and the fourth aG_1_01. All three anibodies were injected at 4 mg/kg. The scale bar (+) represents 20 µm. Below each image is an enlarged view of an NMJ with a scale bar (++) of 10 µm

None of the monoclonal anti-AChR antibodies interfered with cholinergic neurotransmission in the hiPSC model, even at a 100-fold higher concentration than needed to achieve maximum binding to AChR-transfected HEK cells (Fig. 3a). Only two antibodies, aG_1_01 and bG_3_01, mediated complement activation in vitro (Fig. 3b), and neither was as potent as the positive control antibody. None of the four antibodies isolated from patient MG1 were capable of inducing complement activation, even though all of them showed strong specific binding, and the donor's serum was potent in the same complement assay (Fig. 3b, Supplementary Fig. 3).

We examined the pathogenic potential of the two rat-cross-reactive, patient-derived, AChR-binding antibodies in a passive transfer MG model. The cross-reactive antibodies aG_1_01 and bG_4_02 were administered to Lewis rats and the animals’ health and motor performance was evaluated over the course of 48 h, in comparison with rats injected with PBS vehicle, or a control human-derived IgG_1_ (8B4, specific for influenza hemagglutinin). All rats remained healthy, and none showed a significant decline in motor performance as measured by rotarod, compared to animals given vehicle control or the control antibody (Fig. 3c). Histological examination revealed elevated C3 deposition in muscles of animals given aG_1_01, but not in any of the other three groups (Fig. 3d), as was seen in vitro (Fig. 3b). We also confirmed human IgG deposition at the neuromuscular junction in rats injected with aG_1_01 or bG_4_02, and its absence in animals given the vehicle control or the control antibody (Fig. 3e).

### Combining antibodies enhances complement activation synergistically

In view of the observation that serum MG1 induced strong AChR-dependent complement activation (Supplementary Fig. 3), but the four individual anti-AChR monoclonal antibodies isolated from the same donor did not (Fig. 3b), we hypothesized that complement activation is dependent on the synergistic effect of multiple antibodies combined.

**Fig. 4:**
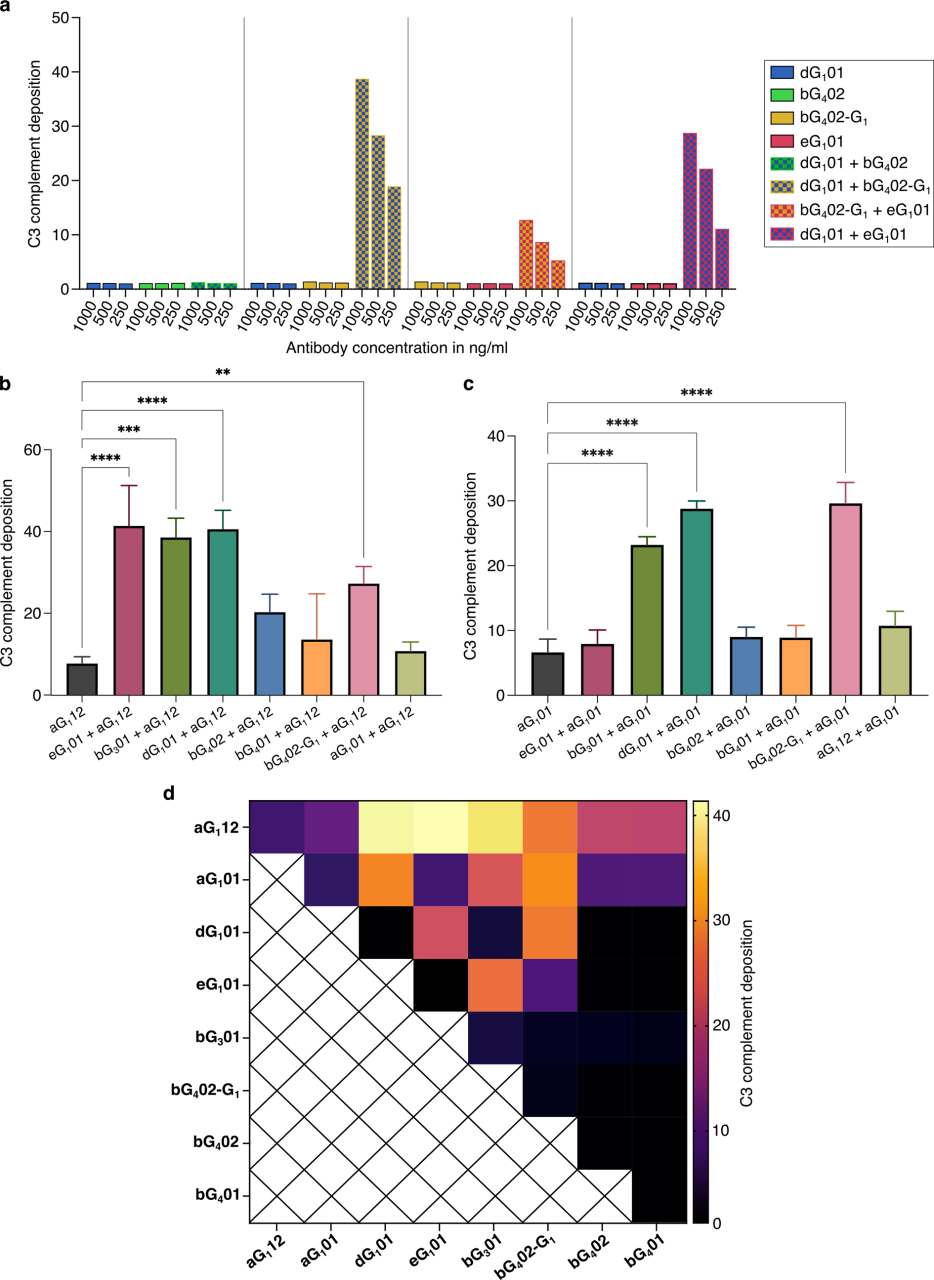
Influence of antibody combinations on complement activation. **a** C3 deposition measured for three different concentrations of AChR-specific monoclonal antibodies, singly or in combination. The concentrations shown on the horizontal axis are the total amount of antibody used, e.g., the very first bar shows the results for 1000 ng/ml of dG_1_01, and the 7th bar 500 ng/ml of each = 1000 ng/ml total antibody. The vertical axis shows the GMFI ratio of C3 immunofluorescence as described in Fig. 1f. Statistical comparison of the ability of single anti-α monoclonal antibodies to activate complement, compared with combinations of these antibodies with other monoclonals targeting different subunits. **b** Shows results for aG_1_12, and **c** the analogous results for aG_1_01. Differences between singles and combinations were analyzed by one-way ANOVA followed by Dunnett’s test (**p < 0.01, ***p < 0.001, ****p < 0.0001). Bars show means of three values from three independent experiments, each with a single measurement for each antibody or combination. Error bars show standard deviations. **d** Heatmap comparing C3 deposition induced by single AChR-specific monoclonal antibodies, and by all possible combinations. Measurements were repeated in three independent experimental runs with a total antibody concentration in each assay of 500 ng/ml

The combination of dG_1_01 and eG_1_01, each at half the concentration (i.e., same final concentration as the previously tested single antibodies), and each with negligible ability to activate complement alone, induced strong complement activation (Fig. 4a).

We tested all possible binary combinations of the patient-derived monoclonal antibodies, and added a well-characterized anti-α IgG_1_ isolated by Makino et al. [25], known to be pathogenic and able to activate complement. We observed combination-dependent complement activation of varying magnitude, from minimal activation similar to the single antibodies, up to a 40-fold increase over baseline (Fig. 4b). The bG_4_02 antibody, originally isolated as an IgG_4_, was additionally tested after artificial class switch to IgG_1_, to enable examination of the effect of different epitope specificities, independent from the constant regions.

Combinations of antibodies with differing AChR-subunit specificity seemed to be required to produce a synergistic effect on complement activation. The two combinations of two antibodies targeting the same subunit i.e., bG_3_01 + bG_4_02-G_1_, and aG_1_12 + aG_1_01, did not result in enhanced complement activation (Fig. 4b–d and Table 2). The strongest enhancement of complement activation was seen in combinations of the α-binding antibody aG_1_12 with antibodies targeting one of the other three subunits (Fig. 4b).

Consistent with the well-known inability of IgG_4_ to activate complement [3, 27] combinations involving the IgG_4_ bG_4_02 showed less complement activity than the analogous combinations with the class-switched derivative bG_4_02-G_1_ (Fig. 4b, c). However, in the context of combinations with an IgG_1_ targeting the α subunit, for example bG_4_01 + aG_1_01, or bG_4_02 + aG_1_12, complement activation was if anything higher than the single α-binding antibody, despite the fact that the concentration of complement-interacting IgG_1_ antibody was halved (Fig. 4b, c).

We also examined the effect of combining antibodies on direct receptor antagonism. No effect on this parameter was detected (Supplementary Fig. 5).

### Autoantibody combination is pathogenic in vivo

**Fig. 5:**
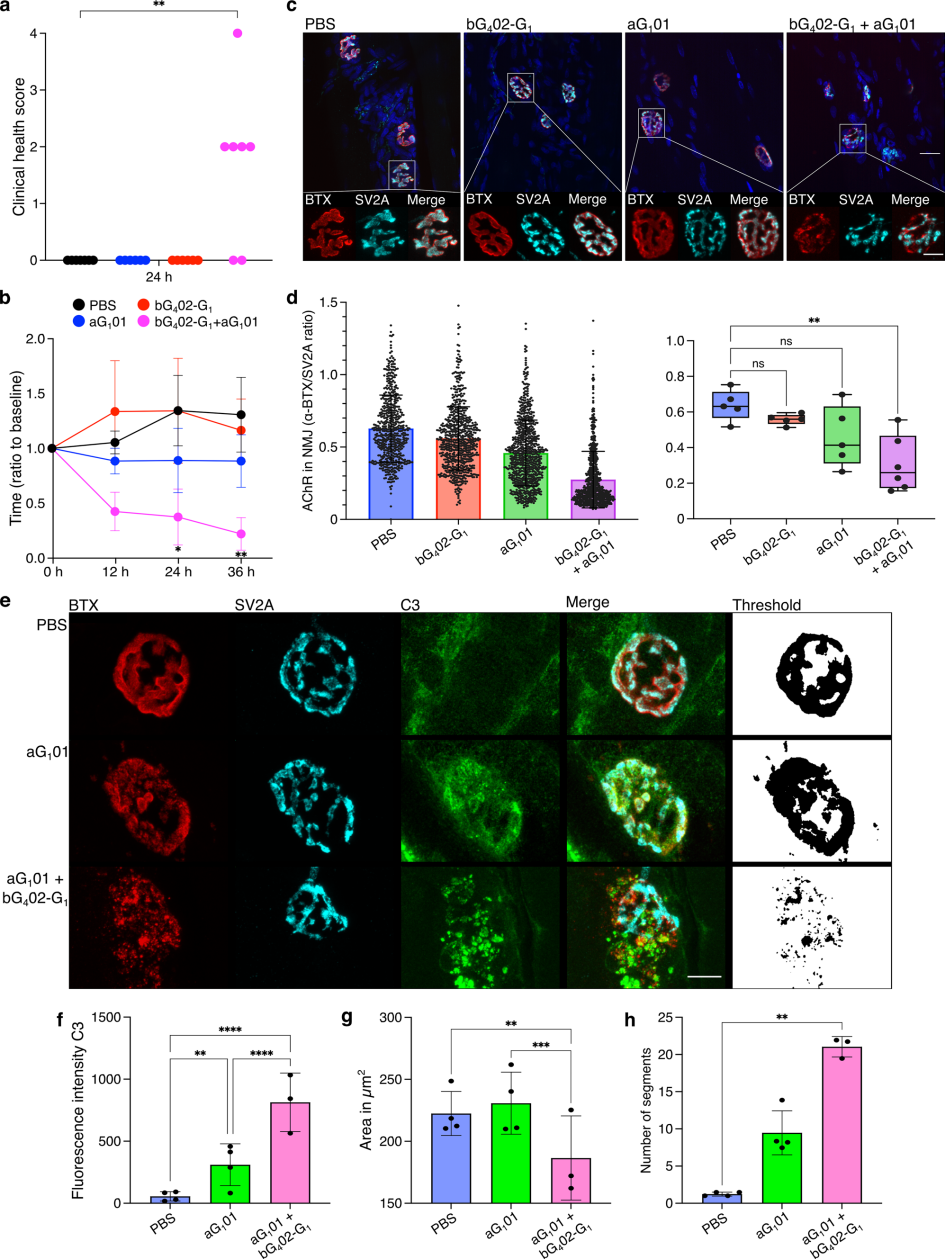
Influence of antibody combination on pathogenicity in a PTMG rat model. **a**–**d** The effect of two single, patient-derived, AChR-specific, monoclonal antibodies, was compared with the combination of both. Female Lewis rats were injected with PBS (*n* = 7), 4 mg/kg aG_1_01 (*n* = 6), 4 mg/kg bG_4_02-G_1_ (*n* = 6), or the combination of 2 mg/kg of each (*n* = 7). bG_4_02-G_1_ is an IgG1, prepared by switching bG_4_02 to IgG_1_ to avoid the confound of different classes. **a**. Clinical scores ranging from 0 to 4 (0 = healthy, 4 = dead, see “Materials and methods”) at 24 h after injection. Differences between each condition and PBS were subjected to Kruskal–Wallis test, followed by Dunn’s multiple comparison (**p = 0.0028). **b** Rotarod performance was tested every 12 h over 36 h following injection, as shown on horizontal axis. The vertical axis shows the relative time animals remained on the rod (ratio of time on rod at measurement, divided by average time for the three trials before antibody administration). Each point displays the mean of all animals tested (6–7 animals per group, pooled from two independent experiments) and error bars show standard error of the mean. If indicated, the differences between antibody-injected and vehicle control groups were signifcantly different (*p = 0.0439, **p = 0.0089) according to Kruskal–Wallis test, followed by Dunn’s multiple comparison. **c** Deterioration of NMJ following monoclonal antibody administration. Cryosections from gastrocnemius muscles from the animals whose behavior is shown in **a**, **b**, sacrificed at 36 h after injection, were labeled with DAPI (blue) to visualize nuclei, α-BTX (red) to visualize AChR, and immunolabeled for SV2A (cyan) to visualize the presynaptic side of the synapse. The four images left to right come from animals given PBS, aG_1_01, bG_4_02-G_1_, and aG_1_01 + bG_4_02-G_1_. Below each image is a series of enlarged images of a single NMJ showing the α-BTX, the SV2A, and the overlay. Scale bar = 10 µm. **d** Relative amount of AChR per NMJ in each of the conditions. The ratio of α-BTX to SV2A fluorescence intensity within the NMJ was calculated for approximately 100 NMJs per animal and each NMJ is represented by one dot on the column scatter plot on the left. The vertical axis shows this ratio. The box and whisker plot on the right shows mean values for each animal, and differences between conditions were subjected to one-way ANOVA followed by Dunnett’s test to compare each condition with PBS (**p = 0.0013). **e–h** Similar experiment with shorter duration to enable histological evaluation of complemen activation at NMJ. **e** Representative high magnification confocal images of rat gastrocnemius muscle cryosections from animals injected with PBS (upper row, *n* = 4), aG_1_01 (middle row, *n* = 4) or the combination of aG_1_01 and bG_4_02-G_1_ (lower row, *n* = 3). Cryosections were labeled with α-BTX, and immunolabeled for SV2A and C3. The monochrome images to the right of the figure show examples of thresholded images based on the fluorescence intensity of the α-BTX label which were used for the structural analysis shown in **g**–**h**. Scale bar = 10 µm. **f** Fluorescence intensity of immunolabeled C3 deposition localized at the NMJ. Results of at least 100 NMJs analyzed per animal are summarized. Differences between experimental groups were statistically significant as indicated (**p = 0.0031, ****p < 0.0001) using a two-way ANOVA with a multiple comparison corrected according to Dunnett. One animal of the cohort injected with the antibody combination was excluded from analysis because serum human IgG measurement was negative. **g** Total NMJ size measured as area labeled by α-BTX. **h** Disintegration of NMJs expressed as number of fragments per NMJ. Single data points in g-h display the means of 15 analyzed NMJs per animal. Differences between groups were subjected to Kruskal–Wallis test with Dunn’s multiple comparison test (**p < 0.005, ***p < 0.0005, ****p < 0.0001)

The discovery that AChR-dependent complement activation by antibodies is so strongly affected by combinations of different epitope specificities raised the question of whether antibody combinations would have a similar impact on in vivo pathogenicity. We, therefore, used a passive transfer rat model to compare the pathogenicity of antibodies administered alone or in combination. We chose the combination of aG_1_01 and bG_4_02 because both recognize the rat receptor as well as human, and neither induced significant complement activation alone. The bG_4_02 antibody was expressed as an IgG_1_ to avoid the confound of comparing antibodies of different classes. The results mirrored the in vitro complement experiments; single antibodies had no significant effect on clinical score or rotarod performance, but the combination of the two antibodies was potently pathogenic (Fig. 5a, b), leading to a significant decrement in rotarod performance by 12 h after injection, and a significant increase in clinical score at 24 h after injection.

To examine the histological processes underlying the behavioral impact of the antibodies, we labeled gastrocnemius muscle cryosections from animals in this experiment with Alexa Fluor 647-conjugated α-bungarotoxin (α-BTX) to visualize the AChR, and antibodies against synaptic vesicle glycoprotein 2A (SV2A) to visualize the presynaptic nerve terminal (Fig. 5c). We delineated the NMJs based on SV2A labeling intensity above an automatically calculated threshold, and within this area measured the ratio of α-BTX signal to SV2A signal. This analysis revealed a significant loss of AChR content on the postsynaptic membrane in rats injected with the combination of both antibodies compared to vehicle controls, or to animals given single antibodies (Fig. 5d).

An obvious question was whether complement activation at the NMJ in vivo would also follow the pattern seen in vitro. At the 36-h time point used for behavioral analysis, the NMJ was already severely degraded, stymying the simple approach of labeling the same tissue for complement analysis. Accordingly, the experiment was repeated with a separate cohort of twelve animals, injected with aG_1_01 alone, or the combination of aG_1_01 and bG_4_02-G_1_, or with PBS vehicle, and sacrificed at an earlier time point (12 h after antibody injection). Cryosections of gastrocnemius muscle were immunolabeled for SV2A and for the complement component C3. AChR at the endplates was visualized with α-BTX (Fig. 5e). First, we quantified the intensity of C3 labeling at individual NMJs in animals in the three conditions. Animals given aG_1_01 alone had significantly more C3 labeling than control treated animals but significantly less than animals given the combination of aG_1_01 and bG_4_02-G_1_ (Fig. 5f). Even at the 12-h time point, detailed structural analysis of single NMJs demonstrated advanced disintegration in animals injected with both antibodies (Fig. 5g, h). The total area occupied by AChR was significantly reduced in animals given the combination of aG_1_01 and bG_4_02-G_1_, while in animals injected with aG_1_01 alone, this parameter was not significantly different from the control group. Overall reduction in AChR-containing area was accompanied by fragmentation of the NMJ in both the single antibody and the combination conditions compared to the vehicle control, but this difference was only statistically significant for the combination condition (Fig. 5h).

### Differential subunit specificity of anti-AChR antibody combinations promotes receptor clustering

**Fig. 6:**
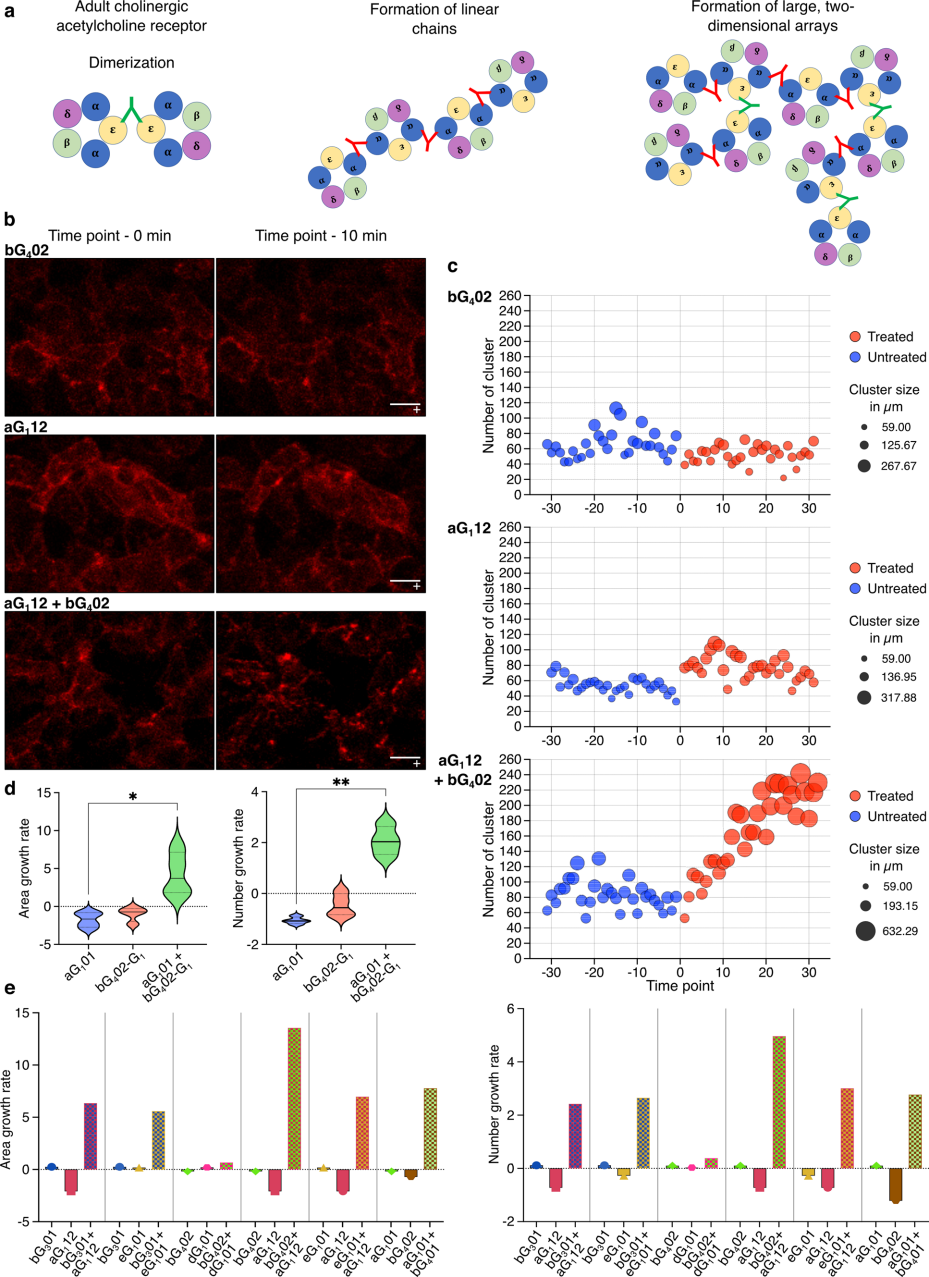
Induction of receptor clustering by anti-AChR antibodies. **a** Hypothetical model of antibody-mediated receptor clustering depending on subunit specificity and combinational synergy. Single, non-α subunit specific antibodies mediate dimerization; α-specific antibodies mediate formation of linear chains; and combinations of antibodies with differing subunit specificity mediate formation of extensive two-dimensional arrays. **b** Live cell imaging of AChR cluster formation induced by antibodies. Images on the left show AChR-transfected HEK cells labeled with α-BTX (red) at time point zero, i.e., after ten minutes of live cell imaging, and before addition of antibody. Images on the right were captured from the same cultures, 10 min after addition of the antibodies indicated to the left of the images, i.e., 4 µg/ml bG_4_02, 4 µg/ml aG_1_12 or the combination of 2 µg/ml of each (4 µg/ml total IgG in each case) scale bar = 10 µm. Supplementary video 2 is a movie compiled from the live cell imaging data shown in the lower row of this figure. **c**. From each image, clusters of AChR, defined as areas of signal exceeding a given threshold were extracted as described in Materials and Methods, and their numbers and sizes plotted as a function of time in the bubble plots on the right. The vertical axis shows the number of clusters, and the size of each bubble corresponds to the average size of the clusters in each image. Each bubble depicts the quantification of one image acquired at one time point during the 20 min of live cell imaging (approximately 3 frames per minute). Blue bubbles represent images taken before, and red bubbles taken after antibody addition. **d, e** Bar graphs showing the growth rate (slope of graph of parameter plotted against time) of either the number or the size of clusters induced by anti-AChR antibodies. **d** Violin plots depicting the growth rates of size and number of clusters of four separately performed experiments for aG_1_01 and bG_4_02-G_1_. The bold line within the violin depicts the median, dotted lines the quartiles. Significant differences between single antibodies and the combinations were assessed by Kruskal–Wallis test with Dunn’s multiple comparison test (*p = 0.0181; **p = 0.0051). **e** Similar information as shown in d, for five combinations of antibodies, as shown below the horizontal axis. Each combination was tested once

Compared with the even distribution over the NMJ in animals given single antibodies, bungarotoxin labeling in animals given the pathogenic combination of antibodies was strikingly focused into patches of 1–10 µm^2^ in area (Fig. 5e). This could be due to direct immune-mediated destructive fragmentation of the postsynaptic membrane, or to antibody-mediated cross-linking of the receptors shown conceptually in Fig. 6a. To investigate this second possibility, we developed a live cell imaging model for examining antibody-mediated receptor clustering in vitro, enabling real-time monitoring of the phenomenon, and avoiding the possible confound of immune destruction.

We transfected HEK cells with AChR and labeled the extracellular domains of the receptor with α-BTX to allow tracking of receptor location by live cell microscopy. The distribution of α-BTX-labeled AChR on the surface of cells was monitored over the course of 10 min before adding either single or combinations of two antibodies, followed by another 10 min of live cell imaging. Single images from before and after antibody addition are shown in Fig. 6b. The formation of large clusters of receptors following antibody-mediated crosslinking can be observed as localized increases in α-BTX signal density, which we tracked as a function of time after antibody addition (Fig. 6c). We tested eight individual antibodies and various combinations. None of the single antibodies had any significant effect on the distribution of the receptor. Combinations of antibodies such as aG_1_12 and bG_4_02, on the other hand, mediated strong clustering (Fig. 6b, c). We investigated this quantitatively for the combination of antibodies (aG_1_01 and bG_4_02-G_1_) used in the in vivo model, and this combination induced a significantly greater increase in the size and the number of AChR clusters compared to single antibodies (Fig. 6d). The artificial class switch had no effect on the phenomenon, since the combination of aG_1_01 and the original bG_4_02 IgG_4_ induced a similar degree of clustering (Fig. 6e). The pattern of strong clustering by the combination, and no effect of the single antibodies were seen in four out of five tested pairs of antibodies, independent of the IgG subclass (Fig. 6e).

## Discussion

The MACACS technique has several advantages over more traditional techniques using labeled soluble antigen. Antigen-specific B cells are strongly enriched because the BCR affinity has to be high enough to extract the membrane antigen from the target cell, and sorting is facilitated because antigen capture leads to an activation of the B cell and CD69 up-regulation [1, 47]. Particularly when the target antigen is a multi-pass membrane protein or protein complex, expression of antigen in live cells enables the isolation of antibodies against a wide variety of epitopes without a priori assumptions about relative epitope importance. This technique allowed us to isolate six monoclonal anti-AChR antibodies of subclasses IgG_1_, IgG_3_ and IgG_4_. Targets included all subunits of the AChR, and all six antibodies showed evidence of affinity-driven hypermutation. We could then investigate whether the pathogenically relevant properties shown by patients' sera could be assigned to specific antibodies.

Single antibodies showed surprisingly little ability to activate complement, or to cause disease in a passive transfer animal model. This changed, however, when antibodies were combined. Combinations of antibodies targeting disparate subunits led to extensive complement activation in vitro, and to a severe behavioral phenotype in vivo. This finding was unexpected, but not completely unprecedented; earlier experiments suggested that rats immunized with a combination of α and β subunits developed myasthenic signs more rapidly than those immunized against α only [19]. Synergy in complement activation between antibodies against different epitopes of single antigens has been observed in the field of anti-cancer antibodies [16], between antibodies against rhesus antigens in hemolytic disease [31], and between antibodies against bacteria [8], but the mechanisms underlying this synergy are not understood. It is well established that isolated IgG molecules are weak inducers of complement, and that some form of multimeric IgG is required to initiate complement signaling [4, 37]. In studies of antibodies interacting with antigen on artificial liposomes, Diebolder et al. (2014) concluded that an optimal complement-activating configuration of IgG is a hexamer of antibodies. According to this model, the individual IgG monomers assemble via interactions between their Fc domains into a wheel-like configuration, with the Fc domains arranged towards the hub, and the Fab domains at the rim. One Fab of each antibody is modeled as binding the antigen, while one Fab arm interacts with the complement C1 component. This configuration would not result in any crosslinking, making it difficult to reconcile with our results, or with the well-established importance of receptor cross-linking in MG [6]. Studies of antibodies interacting with antigens engineered into hexamers with a DNA origami system [33] suggested that the majority of binding configurations involved three antibodies, each bivalently bound to two of the six targets, and not the kind of hexamers described by Diebolder et al. [4]. It is possible that the importance of the hexameric antibody binding configuration is dependent on antigen density. In the NMJ, antigen concentration is very high, with receptor densities estimated in the order of 10,000 receptors per square micrometer [30]. This would put corresponding epitopes on neighboring receptors at distances of the order of 10 nm apart, well within the range of antigen separation for bivalent antibody binding defined by Shaw et al. [33]. We therefore hypothesize that combinations of AChR antibodies are highly complement activating not because they assemble in hexameric configurations, but because they mediate the formation of large, cross-linked lattices of receptor. At the NMJ, the AChR is in some sense "clustered" under normal conditions by the postsynaptic protein rapsyn, under the regulation of a network of other proteins [38]. However, this kind of clustering must be somehow functionally different from that induced by antibodies, since the latter and not the former mediate antigenic modulation [6].

An example of the importance of the two-dimensional supramolecular organization of antigen in the membrane for the activation of complement has been described by researchers working on the aquaporin-4 (AQP4) autoantibody response in neuromyelitis optica [29, 35]. This disease is often associated with autoantibodies against the membrane water channel (AQP4 is expressed in two isoforms, M1 and M23). M1-AQP4 exists as isolated homotetramers in the membrane, while M23-AQP4 tetramers spontaneously assemble into large clusters. Cells transfected with M23-AQP4 are subject to complement-dependent cytotoxicity by patient-derived anti-AQP4 monoclonal IgG, but cells transfected with M1-AQP4 are not [29]. Because this difference is observed even when the cytotoxic antibody binds with similar affinity to the two isoforms, it seems likely that the difference lies in the ability of stable supramolecular platforms of antigen and antibody to act as signal initiation centers for the complement cascade by increasing the local ratio of complement activation versus deactivation by complement regulatory proteins. We propose that in myasthenia gravis, the assembly of AChR into stable two-dimensional arrays that are the substrate of complement-activating antibody clusters is mediated by the cross-linking effect of the antibodies. This requires combinations of antibodies that recognize disparate subunits, as shown in Fig. 6a.

The importance of receptor cross linking rather than antibody density can explain why the addition of a complement-non-activating IgG_4_ can have a positive effect on the degree of complement activation in combination with IgG_1_ targeting other subunits. A clear prediction of the multi-epitope dependency model is that patients whose myasthenic symptoms are caused by complement activation will have autoantibodies against more than one subunit. The subunit specificities of antibodies in patients' sera have been assessed by two methods: by competition with experimental monoclonal antibodies of known subunit specificity [10, 39, 46], and by measuring binding to chimeric receptors containing only one human subunit [23]. Tzartos et al. [43] examined the ability of monoclonal antibodies deemed MIR-, β-, or γ-specific to inhibit binding of antibodies from sera from 86 patients, and concluded that 78%, 20%, and 36% of the 86 sera were inhibited at least 50% by the anti-MIR, anti-β, and anti-γ antibodies respectively. Application of a lower threshold of 10% inhibition would suggest that approximately 99%, 84%, and 95% of sera contain antibodies against the three subunits. These authors detected no correlation between antibody specificity and disease severity.

According to a similar competitive inhibition study by Whiting et al. [46], the least inhibition was exerted by an anti-γ antibody, and the greatest by anti-α, with the anti-β and anti-δ showing intermediate inhibition. These results too, support the conclusion that most patients have antibodies against several subunits. These authors reported "no apparent influence of duration of disease, clinical severity, or previous immunosuppressive treatment over anti-AChR specificity". Heidenreich et al. [11] report inhibition percentages by monoclonals against α, β, γ, and δ, for 20 individual sera. All twenty sera are at least 30% inhibited by antibodies against at least two subunits. These data too, support the prediction that all patients have autoantibodies of multiple subunit specificities.

A corollary to the prediction of multiple specificities is that a person who has antibodies against only one subunit ought to be healthy. Detection of anti-AChR antibodies in non-myasthenia patients is rare, with for example zero positive results out of 427 serum assays from patients with non-myasthenia neurological diagnoses [14]. In summary, the available evidence supports the hypothesis of requirement for multiple subunit specificities for the initiation of MG pathology. This hypothesis predicts that therapeutic approaches designed to interfere with cross-linking [22], especially if combined with a complement-inhibiting strategy [36] are likely to be effective. Also potentially effective in disrupting antibody-crosslinking and receptor clustering might be strategies based on soluble decoy antigens [24].

The antibodies we isolated were, in combination, highly complement activating, but not receptor blocking. Since we did observe direct receptor blockade by sera, we assume that this pathomechanism is relevant, and we expect that future studies with receptor-blocking monoclonal antibodies will be needed to address this. In humans, the therapeutic success of the complement inhibitors eculizumab and ravulizumab in a large proportion of myasthenia patients also points to a pivotal role of complement [13, 26, 45]. While cloning and testing of single recombinant antibodies is not feasible in clinical practice, in vitro testing of sera for their effect on complement activation could provide a tool for assessing this pathway in individual patients. Ultimately, these functional assays might be more meaningful than measuring titers of anti-AChR antibodies in patients.

## Supplementary Information

Below is the link to the electronic supplementary material.

Supplementary file1 (TXT 31 KB)

Supplementary file2 (AVI 42802 KB)

Supplementary file3 (AVI 24581 KB)

Supplementary table 1: List of all patients and healthy controls whose serum was tested for AChR-specific antibody binding, direct receptor antagonism, and complement activation in vitro, as shown in Figs 1a, 1d, and 1f. Supplementary table 2: Genbank flatfile of sequences of variable regions of heavy and light chains of all antobodies cloned (PDF 76 KB)

Supplementary Fig. 1: AChR antagonism by heat inactivated or complement-sufficient serum. The first five sera collected in the course of the project were split into two batches, one of which was heat-inactitated at 56 °C for 30 min before storage at -80 °C for later analysis, and one of which was frozen without heat inactivation. Receptor antagoism by the sera was assayed in the hiPSC-derived neuromuscular system as shown in Main Fig. 1, at three different dilutions as specified above each column scatter plot. Sera from three donors with no neuromuscular disease diagnosis were used as controls. Supplementary Fig. 2: Determination of subunit specificity of anti-AChR antibodies. a Results of studies with rat/human chimeric receptors. Subunits written in black are the rat orthologs, and written in red are human. Figure shows the example of eG101, which is human-specific, and addition of the human epsilon subunit to an otherwise all-rat receptor confers binding. b Results of studies with blocking antibodies. Cells transfected with human AChR were pre-incubated with commercial antibodies of known subunit specificity before adding the patient-derived monoclonals. For example, bG402 is not blocked by an anti-alpha antibody (top right plot) but is blocked by an anti-beta (bottom right plot). Supplementary Fig. 3: Complement activation by individual sera from healthy donors or patients with MG. Supplementary Fig. 4: Immunofluorescence values for anti-C3 labeling at each measured NMJ, pooled from all 16 animals in the experiment shown in Fig. 3d. Supplementary Fig. 5: Direct AChR antagonism by single monoclonal antibodies or combinations of two antibodies. AChR antagonism was measured in the hiPSC neuromuscular system, as shown in Main Fig. 1. The first seven clusters of bars are the same as shown in Fig. 3a. the next two show results from sera from a patient and a healthy donor, and the last three clusters show antibody combinations. Each antibody or combination was tested at 3 concentrations, as shown in the box (TIFF 6457 KB)
