# Supplementary material for: Receptor clustering and pathogenic complement activation in myasthenia gravis depend on synergy between antibodies with multiple subunit specificities
Source: Acta Neuropathol. 2022 Sep 8;144(5):1005–25. doi: 10.1007/s00401-022-02493-6 (PMC9547806; doi:10.1007/s00401-022-02493-6)
Supplement: Supplementary file 4 — Supplementary table 1: List of all patients and healthy controls whose serum was tested for AChR-specific antibody binding, direct receptor antagonism, and complement activation in vitro, as shown in Figs 1a, 1d, and 1f. Supplementary table 2: Genbank flatfile of sequences of variable regions of heavy and light chains of all antobodies cloned (PDF 76 KB) [file 401_2022_2493_MOESM4_ESM.pdf]

Patient ID	Diagnosis	RIA	Sex	Age at date of blood sampling
MG1	Generalized Myasthenia Gravis	17.7	F	57
MG2	Generalized Myasthenia Gravis	5.6	F	74
MG3	Generalized Myasthenia Gravis	800.9	M	46
MG4	Generalized Myasthenia Gravis	850.9	M	45
MG5	Generalized Myasthenia Gravis	16	F	81
MG6	Generalized Myasthenia Gravis	16	F	82
MG7	Generalized Myasthenia Gravis	336	F	40
MG8	Generalized Myasthenia Gravis	10.9	M	81
MG9	Ocular Myasthenia Gravis	11.4	M	65
MG10	Ocular Myasthenia Gravis	51.8	M	78
MG11	Ocular Myasthenia Gravis	2.5	M	73
MG12	Generalized Myasthenia Gravis	0.88	M	73
MG13	Generalized Myasthenia Gravis	41.8	M	69
MG14	Generalized Myasthenia Gravis	336	F	39
MG15	Generalized Myasthenia Gravis	27.4	M	35
MG16	Ocular Myasthenia Gravis	16.5	M	63
MG17	Ocular Myasthenia Gravis	1.9	M	69

Donor ID	Sex	Age at date of blood sampling
HV1	F	29
HV2	F	63
HV3	M	32
HV4	M	29
HV5	M	60
HV6	F	31
HV7	F	23
HV8	F	54
HV9	M	50
HV10	M	65
HV11	F	54
HV12	M	30
