# Supplementary figures and images for: Receptor clustering and pathogenic complement activation in myasthenia gravis depend on synergy between antibodies with multiple subunit specificities
Source: Acta Neuropathol. 2022 Sep 8;144(5):1005–25. doi: 10.1007/s00401-022-02493-6 (PMC9547806; doi:10.1007/s00401-022-02493-6)

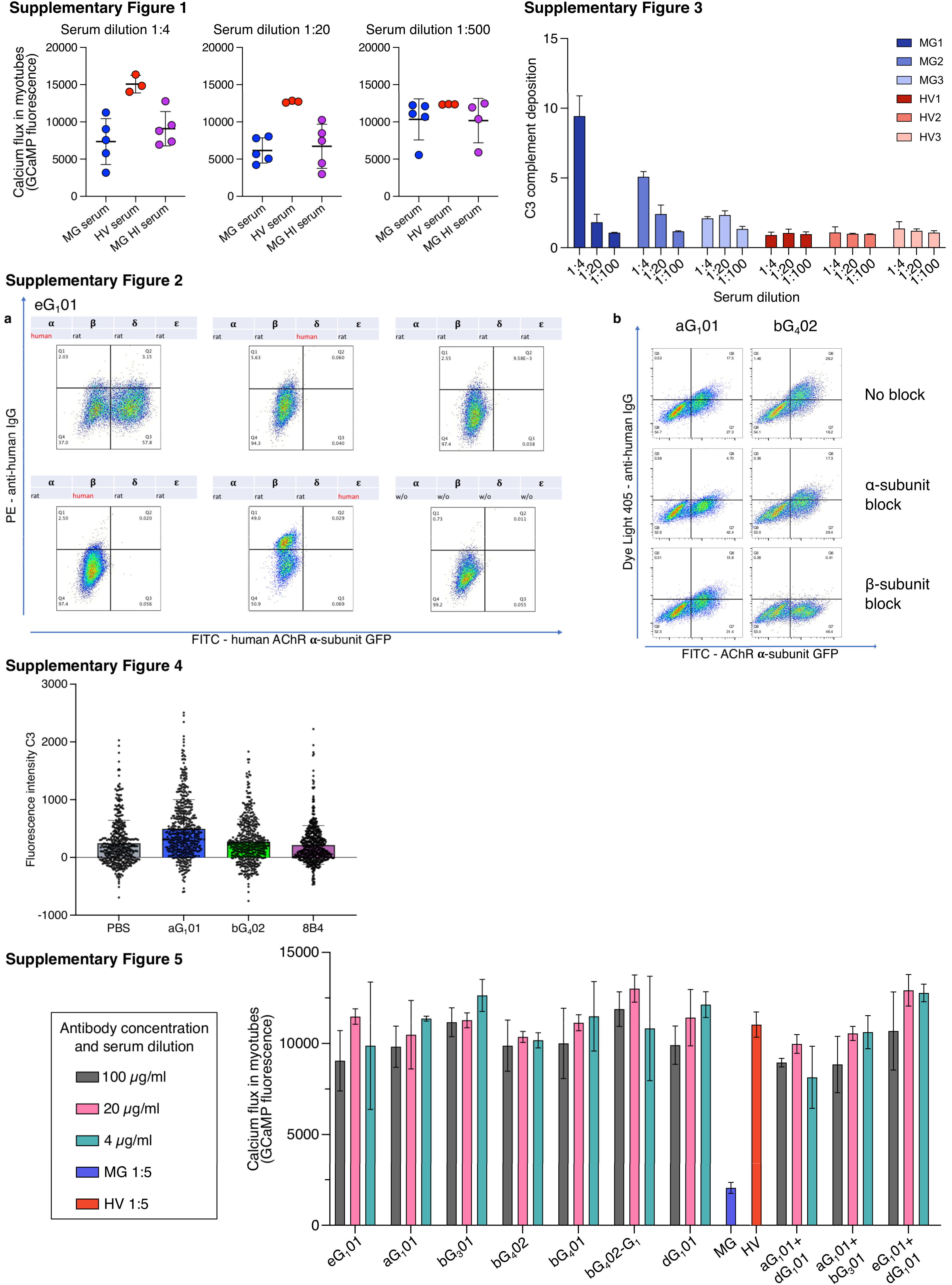

Supplement: Supplementary file 5 — Supplementary Fig. 1: AChR antagonism by heat inactivated or complement-sufficient serum. The first five sera collected in the course of the project were split into two batches, one of which was heat-inactitated at 56 °C for 30 min before storage at -80 °C for later analysis, and one of which was frozen without heat inactivation. Receptor antagoism by the sera was assayed in the hiPSC-derived neuromuscular system as shown in Main Fig. 1, at three different dilutions as specified above each column scatter plot. Sera from three donors with no neuromuscular disease diagnosis were used as controls. Supplementary Fig. 2: Determination of subunit specificity of anti-AChR antibodies. a Results of studies with rat/human chimeric receptors. Subunits written in black are the rat orthologs, and written in red are human. Figure shows the example of eG101, which is human-specific, and addition of the human epsilon subunit to an otherwise all-rat receptor confers binding. b Results of studies with blocking antibodies. Cells transfected with human AChR were pre-incubated with commercial antibodies of known subunit specificity before adding the patient-derived monoclonals. For example, bG402 is not blocked by an anti-alpha antibody (top right plot) but is blocked by an anti-beta (bottom right plot). Supplementary Fig. 3: Complement activation by individual sera from healthy donors or patients with MG. Supplementary Fig. 4: Immunofluorescence values for anti-C3 labeling at each measured NMJ, pooled from all 16 animals in the experiment shown in Fig. 3d. Supplementary Fig. 5: Direct AChR antagonism by single monoclonal antibodies or combinations of two antibodies. AChR antagonism was measured in the hiPSC neuromuscular system, as shown in Main Fig. 1. The first seven clusters of bars are the same as shown in Fig. 3a. the next two show results from sera from a patient and a healthy donor, and the last three clusters show antibody combinations. Each antibody or combination was tested a [file 401_2022_2493_MOESM5_ESM.tiff]
